# Auditory Stimuli Coding by Postsynaptic Potential and Local Field Potential Features

**DOI:** 10.1371/journal.pone.0160089

**Published:** 2016-08-11

**Authors:** Juliana M. de Assis, Mikaelle O. Santos, Francisco M. de Assis

**Affiliations:** Department of Electrical Engineering, Federal University of Campina Grande, Campina Grande, Paraíba, Brazil; McGill University Department of Physiology, CANADA

## Abstract

The relation between physical stimuli and neurophysiological responses, such as action potentials (spikes) and Local Field Potentials (LFP), has recently been experimented in order to explain how neurons encode auditory information. However, none of these experiments presented analyses with postsynaptic potentials (PSPs). In the present study, we have estimated information values between auditory stimuli and amplitudes/latencies of PSPs and LFPs in anesthetized rats *in vivo*. To obtain these values, a new method of information estimation was used. This method produced more accurate estimates than those obtained by using the traditional binning method; a fact that was corroborated by simulated data. The traditional binning method could not certainly impart such accuracy even when adjusted by quadratic extrapolation. We found that the information obtained from LFP amplitude variation was significantly greater than the information obtained from PSP amplitude variation. This confirms the fact that LFP reflects the action of many PSPs. Results have shown that the auditory cortex codes more information of stimuli frequency with slow oscillations in groups of neurons than it does with slow oscillations in neurons separately.

## Introduction

It is a fact well known in neuroscience that the brain is an organ with enormous computational capabilities. It accomplishes a huge number of sensory activities, regulates our thoughts, emotions and behavior [1]. However, it remains a mystery how brain computation is done. This has been the subject of various works on neural encodings and decodings [2] [3] [4] [5] [6] [7] [8]. In this context, the term *encoding* stands for the process of transforming experimentally observable sensory stimuli into neural responses. The term *decoding* stands for the process of using neural responses to reconstruct features of the original stimulus [9]. A method which is usually adopted in the study of sensory information processing involves the stimulation of the sense organ of interest with physical stimuli and the consequent observation of all responses produced by a selected part that is being measured [10]. Neural encodings have been explored in both human and animal sensory cortex, including the visual, the auditory, the somatosensory, and the gustatory cortices [2] [3] [4] [5] [6] [7] [8]. Encodings are usually done with spikes or local field potentials (LFPs). The former are obtained from high-frequency (> 500Hz) filtered signals electrophysiologically registered in the brain and the latter are derived from low-frequency registered (< 500Hz) [11] [12]. Spikes are important elements in rate and temporal codes. Rate codes determine how many spikes are observed in a number of trials or at different time windows in order to compute a mean. Temporal codes, however, use the precise time relations between spikes of distributed neurons [13]. Codes using local field potentials (LFPs) generally use LFP power and phase at different frequency bands, either to estimate sensory information or to improve spike codes [2] [3] [4].

To assess the efficiency of neural encodings in brain computations, mutual information—a concept introduced in information theory [14]—is commonly used. Other concepts introduced by information theory are equally valuable to neuroscience, such as data processing inequality and directed information [5] [15] [16]. Mutual information is not an exclusive measure for neural codes, but it has been extensively used along with sensory information encoding [2] [3] [4] [5] [6] [7] [8]. Despite all these attempts to evaluate encodings of sensory stimuli into several neurophysiologic responses, performed in different sensory cortices, we did not find any work that has analyzed or measured information levels with postsynaptic potentials (PSPs), especially in the auditory cortex.

In the present study, PSP refers to the invasive register obtained by whole cell patch-clamp recording to measure the difference in the membrane electrical potential derived from the net influx of ions in the neuron. The measured PSP in one neuron may result from more than one synapse, whose integrated changes in postsynaptic membrane electrical potential may generate a spike [17]. Thus, PSP registers the electrophysiological changes in each neuron separately. In this sense, they are similar to spike trains. These can indicate the activity of neurons individually [18]. On the other hand, it is widely accepted that LFPs will result from the subthreshold action (such as PSPs) of many neurons, even though it is not easy to establish how many of these are involved [19] [20].

In such context, the present paper attempts to investigate the achievable values of PSP coding in contrast with the achievable values of LFP coding in bits. We have estimated which register produced more auditory information as a mean to identify if the coding was better implemented, observing, at the same time, the action of neurons either individually (PSPs) or collectively (LFPs). With this purpose in mind, we have chosen the main features of PSP and LFP registers—amplitudes and latencies—and have calibrated a method which can be more accurate in producing information estimates than those obtained by the traditional binning method. In addition to these information estimates, we present an analysis on how the different anesthesia, used in animals under experiment, affects information estimates.

## Materials and Methods

### Experimental Data

The data is available at http://crcns.org/ a data sharing website, created to promote useful and trustful data sharing among computational neuroscience researchers [21] [22]. The methods are fully explained in the reference [23]. Michael DeWeese, working at Anthony Zador’s laboratory, registered the data in order to evaluate variability in tone-evoked responses. DeWeese presented 32 different frequency tones, logarithmically spaced between 2kHz and 46,731Hz, with the same amplitudes and the same duration (25ms) to anesthetized rats. The multiple repeated presentation of tones had a pseudo-random order, at a 2 *per* second fixed rate. DeWeese registered PSPs with *in vivo* whole cell patch-clamp, and LFPs with a second nearby (≈ 0.5mm) patch electrode (simultaneously). DeWeese registered both PSPs and LFPs on different parts of the primary auditory cortex (A1) of the rats, yet in the same cerebral hemisphere. The sampling rate for both PSP and LFP recordings was 4000 samples/s. DeWeese used about 17 animals (DeWeese, personal communication) and recorded 33 cells. The anesthesia employed for 16 of the cells was pentobarbital (65mg/kg). Diazepam (5mg/kg) was also used in 3 of these 16 cells. In the remaining 17 cells, urethane (1.5g/kg) was used after surgery performed under ketamine (60mg/kg) and medetomedine (0.5mg/kg). Data were registered at Cold Spring Harbor Laboratory, New York, U.S., in accordance to National Institutes of Health guidelines, and approved by Cold Spring Harbor Laboratory Animal Care and Use Committee.

### Information Theoretic Analysis

Information theory is a relatively new science consolidated by Claude Shannon in a publication of 1948 [24]. One of the most used concepts of information theory applied to neuroscience includes mutual information (MI), which assesses how uncertainty of a random variable *S* is diminished by the knowledge of another random variable *R*, as seen in Eq (2), for discrete random variables *S* and *R*, where *H*(*S*) is the entropy (or uncertainty) of *S* and *H*(*S*|*R*) is the entropy of *S* when *R* is known [14].

$$I\left(S;R\right)=\sum_{S}\sum_{R}p\left(s,r\right)\log\frac{p(s,r)}{p(s)p(r)}$$ (1)

=H(S)-H(S|R). (2)

When the logarithm in Eq (1) is base 2, MI is given in bits. When the natural logarithm is used, MI is given in nats.

MI shows how far from independent two random variables are. This is most applicable in neuroscience because it establishes a general measure to evaluate the coding between sensory stimulus, *S*, and neurophysiological responses, *R*. Besides, MI is more reliable than Pearson correlation coefficient, in the sense that it captures nonlinear categories of correlation. Mutual information is zero if and only if the variables are independent. It is possible, however, that random variables expressing zero Pearson correlation are actually dependent [25] [26].

In this paper, the random variables correspond to the frequency of the stimulus presented discretely in 32 different frequency tones with the same amplitude and duration along with a continuous feature of neurophysiological responses. MI between a discrete random variable and a continuous random variable can be evaluated as in Eq (3), where *μ*(*s*, *r*) is the joint distribution of *S* and *R*, *p*(*s*) is the marginal distribution of *S*, and *μ*_*R*_(*r*) is the marginal distribution of *R*. Nevertheless, it can be very difficult to estimate MI from data whose distribution is unknown.

$$I\left(S;R\right)=\sum_{s}\int \mu \left(s,r\right)\log\frac{\mu (s,r)}{p\left(s\right)\mu_{R}\left(r\right)}dr$$ (3)

The binning method is commonly used, especially in neuroscience, to group the continuous data into bins. After this, a naive estimator will compute the occurrence of each bin response and each stimulus. Then relative frequencies will be used as probabilities in the Eq (1). These will represent the maximum likelihood estimators for probabilities [27]. However, it is commonly known that, by using the naive estimator, biased estimates will be obtained. A method which is most used to correct this bias is the quadratic extrapolation method (QE) [28] [29] [30] [31]. QE method consists in dividing the samples into random partitions with 1/2 and 1/4 of the original data, computing the information naive estimation of the subgroups and then computing the mean for the 2 and the 4 values. The pairs (*N*, *I*_*naive*1_(*S*;*R*)), (*N*/2, *I*_*naive*2_) and (*N*/4, *I*_*naive*4_) are used to fit a curve and to find the parameters *a*, *b* and *I*_*true*_:

$$\begin{array}{lll} I_{naive1}\left(S;R\right) & = & I_{true}\left(S;R\right)+\frac{a}{N}+\frac{b}{N^{2}},\\ I_{naive2}\left(S;R\right) & = & I_{true}\left(S;R\right)+\frac{a}{\left(N/2\right)}+\frac{b}{{\left(N/2\right)}^{2}},\\ I_{naive4}\left(S;R\right) & = & I_{true}\left(S;R\right)+\frac{a}{\left(N/4\right)}+\frac{b}{{\left(N/4\right)}^{2}}, \end{array}$$

where *I*_*true*_ is the true information estimate and *N* is the size of the data set.

Despite promoting some improvement over naive estimation, QE proved to be inefficient in our simulations to avoid bias. In order to calibrate an information estimation method, we generated data sets with analytically known mutual information for two simple examples demonstrating the weakness of the binning method and considering its bias correction in order to make MI estimates. We generated *S* a discrete random variable assuming values 1 or 2 with equal probability. Moreover, *R* exhibited the following conditioned probability on *S* Eq 4:

$$p\left(R|S\right)=\left\{\begin{array}{c} \frac{1}{2}{\text{ret}}_{2}\left(r-1\right),\;s=1,\\ \frac{1}{2}{\text{ret}}_{2}\left(r-2\right),\;s=2, \end{array}\right.$$ (4)

where ret_2_ is the rectangular pulse with width 2, centered in zero. The true mutual information is 0.25 bit.

Based on reference [33], Ross proposes a method to estimate MI between discrete and continuous random variables [32]. The method uses, for each data point *i*, the distribution of the *k*^th^-nearest neighbour distances for a given value of the discrete variable (Eq (5)):

$$I_{i}=\psi \left(N\right)-\psi \left(N_{S_{i}}\right)+\psi \left(k\right)-\psi \left(m_{i}\right).$$ (5)

*N* is the size of the data set, *N*_*S*_*i*__ is the number of points for which the discrete variable is *S*_*i*_, *k* is the chosen number of neighbors, and *ψ* is the digamma function. The term *m*_*i*_ corresponds to the number of points at a distance given by the *k*^th^-neighbour of *i* among the points *N*_*S*_*i*__. MI estimate is obtained by calculating the average of *I*_*i*_ over all data points:

$$I=\langle I_{i}\rangle$$ (6)

**Fig 1:**
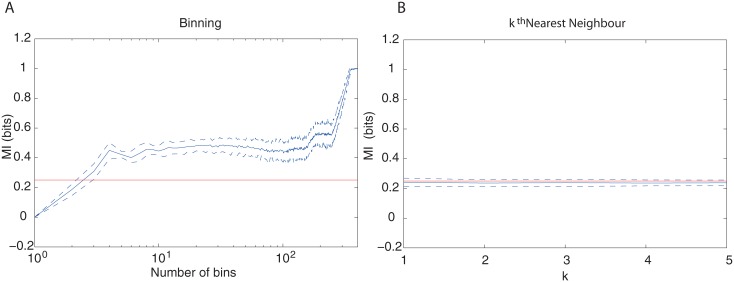
Simulation for uniform conditioned probability. (A) MI estimation using the binning method with QE correction, as a function of the number of bins used. (B) MI estimation using the method of the *k*^th^ nearest neighbours, as a function of the chosen *k* value. Sample size was *N* = 400, red line indicates the true value of MI, blue line indicates mean MI value for 100 data sets of size 400 each. The interval between dashed blue lines indicates 10% lowest to 10% highest estimates.

Fig 1 compares the two methods. For the binning method, results were obtained by varying the number of bins from 1 to 400, and by using the QE correction. It is clear that, despite its simplicity, the binning method depends strongly on the number of bins, and for most bins used, this is still inaccurate. However, the second method reveals estimates much closer to the actual value of MI.

**Fig 2:**
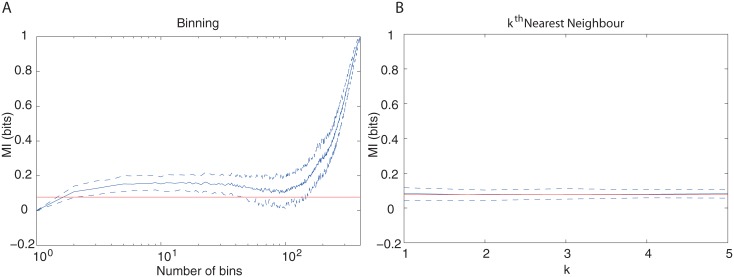
Simulation for Gaussian conditioned probability. (A) MI estimation using the binning method with QE correction, as a function of the number of bins used. (B) MI estimation using the method of the *k*^th^ nearest neighbours, as a function of the chosen *k* value. Sample size was *N* = 400, red line indicates the true value of MI, blue line indicates mean MI value for 100 data sets of size 400 each. The interval between dashed blue lines indicates 10% lowest to 10% highest estimates.

The second example is shown in Fig 2, revealing again the superiority of the method of the *k*^th^-nearest neighbour. Here, *S* is a discrete random variable assuming values 1 or 2 to be of equal probability. *R* has the following conditioned probability on *S*:

$$p\left(R|S\right)=\left\{\begin{array}{c} \frac{1}{\sqrt{2\pi }}e^{-\frac{{\left(r-1\right)}^{2}}{2}},\;\text{if}\;s=1,\\ \frac{1}{\sqrt{2\pi }}e^{-\frac{{\left(r-2\right)}^{2}}{2}},\;\text{if}\;s=2. \end{array}\right.$$ (7)

The true mutual information is 0.08 bit.

Although the estimates vary according to the chosen value of *k* neighbors, in the second method of estimation this variation is small when compared to the one that depends on the number of bins, which would happen when applying the binning method. Here, we can choose *k* = 3 [33]. Moreover, in both examples, the estimates produced by the second method are much closer to the theoretical value.

### Filtering and Features Attainment

**Fig 3:**
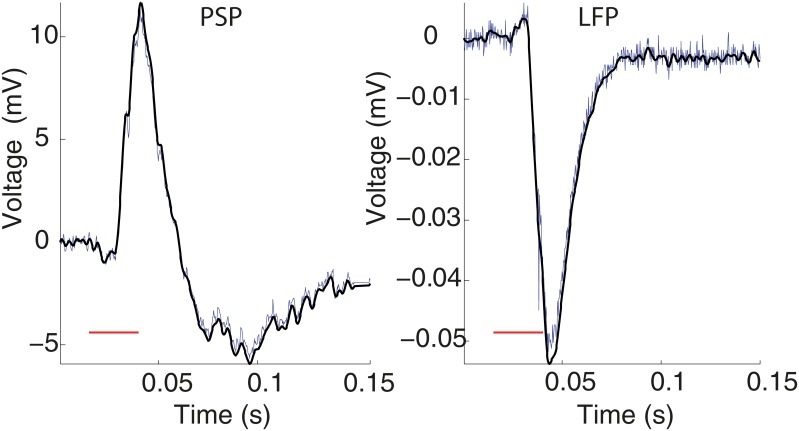
Filtering. PSP and LFP registers with and without low-pass filtering, 300Hz (black and blue curves, respectively). Examples from cell 13 (030201md03b) in response to 5527Hz. Electric potential relative to baseline, mean potential in first 15ms. Red trace indicates stimulus presentation.

**Fig 4:**
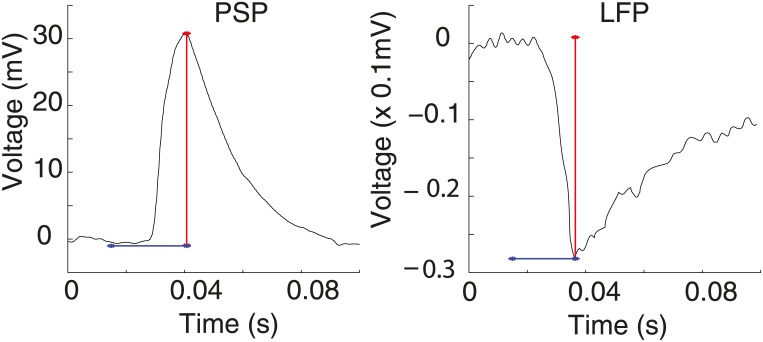
Chosen features. Features from cell 12 (030201md01b), in response to 3003Hz: blue latency and red amplitude.

The data were low-pass filtered (300Hz) for both PSPs and LFPs registers, see Fig 3. The filter used a two-way least-squares finite impulse response (FIR) filtering and its order was 39 (we used the eegfilt.m routine in *Matlab* from the EEGLAB toolbox; Delorme and Makeig 2004 [34]). This procedure is plausible, since both PSP and LFP registers have only slow waves components. The continuous features of the neurophysiological responses were then obtained from the time interval of 15ms, stimulus presentation, to 100ms. Fig 4 shows some examples of the resulting features. These features are amplitudes taken from minimum to maximum and latencies, taken from stimulus presentation up to the time the curve reaches its maximum in the PSP case, or its minimum, in the LFP case.

## Results

This section presents the estimates set up by the chosen estimator and data features. The mean values and standard error measure (SEM) for the MI estimates in bits were (mean ± SEM): 0.12±0.02, 0.09±0.02, 0.16±0.02, and 0.11±0.02, for PSP amplitude, PSP latency, LFP amplitude and LFP latency cases, respectively.

**Fig 5:**
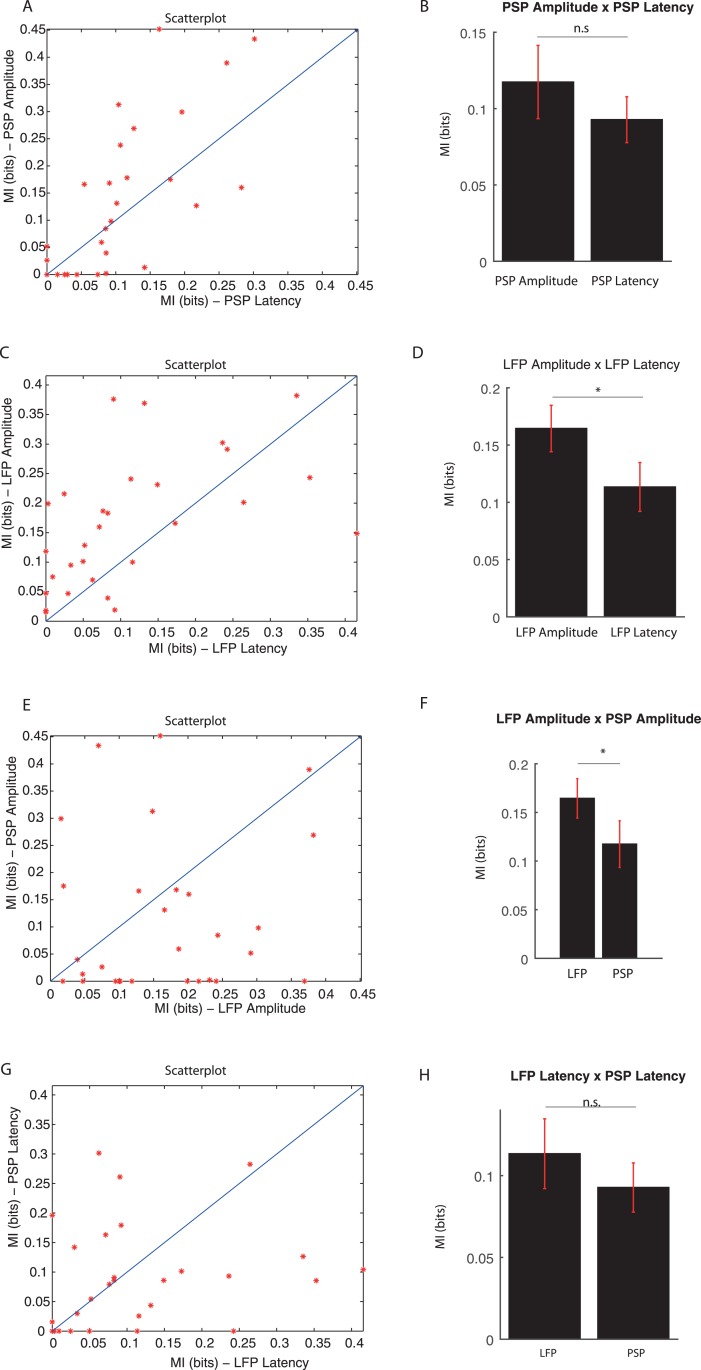
MI estimates from PSP latencies, PSP amplitudes, LFP latencies and LFP amplitudes, in bits. (A, C, E, G) Scatterplots for PSP amplitude MI versus PSP latency MI, LFP amplitude MI versus LFP latency MI, PSP amplitude MI versus LFP amplitude MI, PSP latency MI versus LFP latency MI, respectively. (B, D, F, H) Bar graphs for MI means, with SEM in red lines, for each case corresponding to scatterplots of (A, C, E, G), respectively. According to Wilcoxon one-tailed test, there were no significant differences between PSP amplitude and PSP latency MI estimates (*p* = 0.108), and between PSP latencies and LFP latencies MI estimates (*p* = 0.159). However, according to results obtained by this same test, there have been observed significant differences between LFP amplitude and LFP latency MI estimates (*p* = 0.003), and between LFP amplitude MI and PSP amplitude MI estimates (*p* = 0.026).

The first information analysis is carried out on each type of signal, PSP or LFP, focusing on the most informative feature, amplitude or latency. The results for the PSPs are shown in Fig 5, panels (A) and (B), while the results for the LFPs are shown in panels (C) and (D).

Wilcoxon signed-rank one-tailed tests indicated that amplitudes and latencies from PSPs are equally informative (*p* = 0.108); whereas, in the LFP case, information in amplitudes is significantly greater than information in latencies (*p* = 0.003). Significance level is set in 5% in all statistical tests performed.

The next analysis was performed following the same features of different signals, i.e., it resulted from comparisons between MI from PSP amplitudes and LFP amplitudes as well as from comparisons between MI from PSP latencies and LFP latencies. Fig 5 exhibits the amplitude comparison in panels (E) and (F), showing that LFP is more informative than PSP in this feature coding. Wilcoxon signed-rank one-tailed test confirmed this hypothesis (*p* = 0.026). Fig 5 also illustrates the latency comparison, in panels (G) and (H). The Wilcoxon signed-rank test confirmed that estimates from PSP and LFP latencies cannot be set apart from different distributions (*p* = 0.159).

**Fig 6:**
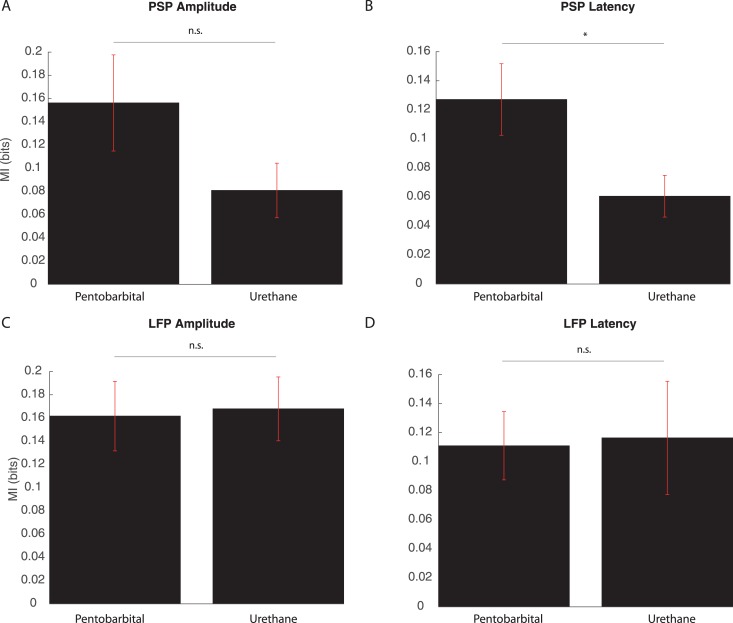
Comparison between MI estimates when different anesthetics were used (pentobarbital and urethane). (A) Means of MI estimates of PSP amplitudes, Wilcoxon rank-sum test indicated no significant difference (*p* = 0.103). (B) Means of MI estimates of PSP latencies, Wilcoxon rank-sum test indicated significant difference (*p* = 0.021) (C) Means of MI estimates of LFP amplitudes, Wilcoxon rank-sum test indicated no significant difference (*p* = 0.388). (D) Means of MI estimates of LFP latencies, Wilcoxon rank-sum test indicated no significant difference (*p* = 0.708). In all cases, red lines indicate SEM.

The last analysis was on the use of different anesthetics. Implications on the MI levels caused by the use of two different drugs—pentobarbital and urethane—were also investigated. For 16 of the cells registered, pentobarbital was used, while for the rest 17 cells, urethane was used as anesthesia. Fig 6 presents the means and SEM for MI estimates of PSP amplitudes, PSP latencies, LFP amplitudes and LFP latencies when the two drugs were used. The values for MI estimates, for cells under the effect of pentobarbital and urethane, respectively, were: 0.16 ± 0.04 bits against 0.08 ± 0.02 bits, for PSP amplitudes; 0.13 ± 0.02 bits against 0.06 ± 0.01 bits, for PSP latencies; 0.16 ± 0.03 bits against 0.17 ± 0.03 bits, for LFP amplitudes and 0.11 ± 0.02 bits against 0.12 ± 0.04 bits, for LFP latencies. The Wilcoxon rank-sum test confirmed that for the cases of PSP amplitudes, LFP amplitudes and LFP latencies there were no differences in the MI estimates with the use of one anesthetic (*p* = 0.103, *p* = 0.388 and *p* = 0.708, respectively). The only case where the one-tailed test showed a significant difference was that of PSP latencies, where the use of pentobarbital presented greater MI values (*p* = 0.021).

## Discussion

We estimated information measures for encoding the transformation of auditory stimuli into features of PSPs and LFPs in anesthetized rats *in vivo*. Therefore, we filtered the recordings and selected the features that would become reduced responses while encoding. These features, called amplitudes and latencies, were related to the power (amplitude) or time (latency) of the signals, and were not discretized.

Before discussing the results, it must be stressed that we have used signedrank Wilcoxon test because this test is broadly applicable and does not impose greater demands on the samples. Indeed, it requires that paired samples be independent, not necessarily coming from normal distributions. However, the test takes into account only the ranking of differences, not the value of the differences themselves [35].

Concerning the main results from the information estimates, it is convenient to know that PSP latencies are as informative as PSP amplitudes. If the coding had been made with spikes, the results might not have been the same as all stimuli come from the same spatial location relative to the rats. This is a factor that changes neuron spike times. However, PSP signals hold their characteristic of being temporally and spatially summed, along dendrites to the soma, in a classical situation. For the PSP time to reach its maximum, one has to relate it to these two summation types. Moreover, the closer temporal or spatially in postsynaptic cells, the two neurotransmitter package releases are, the greater the depolarization of the cell under patch clamp is (if the synapses are excitatory, as the ones registered here). In other words, the greater spatial-temporal summation is, the greater PSP amplitudes and the shorter PSP latencies will be.

Other related factors to PSP information latency are the receptor types in the patch clamp cell. The principal central nervous system excitatory neurotransmitter is the glutamate, which presents two important ionotropic receptors: AMPA and NMDA. AMPA receptor depolarization is faster, while NMDA receptor depolarization is slower and long-lasting relative to the magnesium ion blockade [36]. Therefore, all these variables—number of channels and types—as much as the net organization of neurons, synapses and posterior interference over spatial-temporal PSP summation, are intermediate between the variables of interest, i.e., stimuli and responses. As it is, information estimates were made separately by the cell, which means that it exhibits constant properties (unless there is a possible long-term potentiation or long-term depression mechanism, not considered in the present paper).

Additionally, the LFP amplitudes are more informative than the LFP latencies, and than the PSP amplitudes. This reflects the fact that LFPs come from both gathered neurons activity and their subthreshold activities, like PSPs. The results presented here also indicate that A1 codes more information from slow oscillations from the joint activity of neurons, than it does from the slow oscillations from the activity of neurons, separately.

The last analysis is based on the fact that the anesthetics used did not influence on the information estimates, except for the case of PSP latencies. This shows that the anesthetic used influenced on the delay of each cell’s response. Nevertheless, it remains to be investigated whether the results found are also valid for unanesthetized animals.

Moreover, we should stress that some of the information estimates used with the *k*^th^-nearest neighbour method produced negative results. These values were set to zero in the analysis. These apparently strange results were possible since systematic errors could happen by using the *k*^th^-nearest neighbor method [33]. Therefore, there are neurons that do not code any information about auditory stimuli. Though it is established that memory and learning might modify tonotopic map [37], this was not the case for this experiment, which was not linked to any reward activity. A plausible explanation is that these cells may be related to modulation on other cerebral cortex layers, since auditory cortex activity is submitted to activities of attention or relaxation [38].

In addition to what we have presented, a plausible comparison to other works in this area involves the mathematical modeling of the physical feature being altered, i.e., the stimuli random variable *S*. It has become clear in the present study that the only auditory stimulus that changed each time it is presented is the tone frequency, which is distributed uniformly. However, many papers show a single time window as stimulus, and present this ‘variable’ as uniform because it is repeated the same number of times [4] [3] [2] [6]. Although each time window reflects a singular package of stimuli, other time windows may show similar physical stimuli, regarded as unique simply for being presented in another interval. The physical feature generating a particular neurophysiological response could be the intensity or frequency of a sound or light wave, for example. Its temporal duration is also relevant, but it does not exhibit such oversimplification.

Finally, we stress that the accuracy of the method used to estimate MI is very important in order to produce reliable results and to give consistency regarding the conclusions implied by them. By this we mean that the closer the estimates are to the true values of information, the more confident we will be to state that the feature of a register is more informative than another, or else to examine how the brain manages sensory processing. The *k*^th^-nearest neighbor method differs from the binning method because it does not demand discretization of the continuous variable, it does not depend on the number of bins used (once it does not use bins) and, especially, it gives more accurate results.

## Conclusion

It may be concluded from the present work that LFP amplitudes give more information about the frequency of auditory stimuli than PSP amplitudes. This can be related to the fact that LFP includes subthreshold activity (as PSPs) of many neurons. Thus, slow oscillations from A1 code the information from the frequency of auditory stimuli from neurons collectively. It has been also concluded that the frequency of auditory stimuli encoding with LFP amplitudes gives greater MI values than that with LFP latencies. Besides, PSP amplitudes and latencies bring the same amount of information from auditory stimuli frequency. The present paper demonstrates that MI estimation, obtained by the binning method, can be rather misleading, in spite of bias correction, such as QE.
